# A Case of Hemophagocytic Lymphohistiocytosis in a Patient with Chronic Lymphocytic Leukemia after Treatment with Fludarabine, Cyclophosphamide, and Rituximab Chemotherapy, with Autopsy Findings

**DOI:** 10.1155/2012/326053

**Published:** 2012-12-17

**Authors:** Ing S. Tiong, Michael B. Y. Lau, Seventeen Toumoua, Shingirai Chiruka

**Affiliations:** ^1^Southern Blood and Cancer Service, Dunedin Hospital, Private Bag 1921, Dunedin 9054, New Zealand; ^2^Division of Haematology, Southern Community Laboratories Ltd., Dunedin 9016, New Zealand

## Abstract

Hemophagocytic lymphohistiocytosis (HLH) is rarely described in association with chronic lymphocytic leukemia (CLL), mostly triggered by disease progression or concurrent infection. A 68-year-old male received 4 cycles of fludarabine, cyclophosphamide, and rituximab (FCR) for CLL and achieved a complete response. Twenty-four days after the last chemotherapy, he presented with febrile neutropaenia and was diagnosed with HLH. The diagnosis was based upon persistent fever, pancytopenia, hyperferritinemia, splenomegaly, and hemophagocytosis on bone marrow aspirate. He began treatment with dexamethasone, cyclosporine, and etoposide. Fever resolved and hyperferritinemia improved but pancytopenia persisted. He died 13 days later from septic shock with positive blood cultures. A limited postmortem examination was performed and biopsies were taken from bone marrow, liver, and spleen. Biopsies demonstrated abundant hemophagocytosis by the activated macrophage as stained by CD68. There was no evidence of residual CLL as demonstrated by the lack of lymphocytes which was confirmed by the negative staining of CD79a. Chemotherapy appears to be responsible for the development of HLH in this patient. This is the second reported case of HLH developing after a rituximab-containing chemotherapy.

## 1. Introduction

Hemophagocytic lymphohistiocytosis (HLH) is rarely described in association with chronic lymphocytic leukemia (CLL), mostly triggered by disease progression or concurrent infection. Physicians who care for immunocompromised patients need to recognize this often fatal complication of common infections or malignancies. Chemotherapy agents are rarely directly implicated in the development of HLH.

## 2. Case Presentation

A 68-year-old male was first diagnosed with B-cell chronic lymphocytic leukemia (B-CLL) in July 2006, based on incidental finding of lymphocytosis, blood film morphology, and the typical lymphocyte surface markers with positive CD19, CD5, CD23, and weak expression of surface lambda. His other medical comorbidities include obesity, obstructive sleep apnea, hypertension, stable angina, and erectile dysfunction.

He developed B-symptoms and cytopenias (Binet stage C) in November 2011. Bone marrow biopsy revealed a hypercellular marrow, with an infiltrate of small mature lymphocytes, and reduced but normal morphology and maturation of the erythroid, myeloid, and megakaryocytic lineages. Fluorescence in situ hybridization (FISH) was negative for ataxia telangiectasia mutated (*ATM*) and tumor protein p53 (*TP53*) deletion. Treatment with immunochemotherapy consisting of rituximab (375 mg/m^2^ day 1 first cycle then 500 mg/m^2^ day 1 every 28 days on subsequent cycles), fludarabine (orally 24 mg/m^2^/d day 1–5 every 28 days), and cyclophosphamide (orally 150 mg/m^2^/d day 1–5 every 28 days) was commenced and he completed 4 out of 6 planned cycles in February 2012. The treatment was stopped after 4 cycles due to failure to adequately recover the blood counts. A bone marrow aspirate and trephine biopsy done at this time did not show any residual CLL (i.e., complete remission) and there was mild dysplasia in the erythroid and myeloid lineages consistent with recent chemotherapy. Normal macrophages were noted.

24 days after the last chemotherapy, the patient presented with febrile neutropaenia. He was initially treated with Piperacillin/Tazobactam, later changed to Meropenem and Tobramycin, with Voriconazole added. However, the fever persisted despite the empiric antimicrobial therapy. He did not have any family history of suspicious febrile events, autoimmune disorders, or HLH. During this period, extensive search for infective causes failed to yield a focus: multiple blood and urine cultures for bacteria, mycobacteria and fungi; blood serology for hepatitis B, C, and parvovirus B19; polymerase chain reaction (PCR) for cytomegalovirus (CMV) and Epstein-Barr virus (EBV); nasal swab PCR for rhinovirus, adenovirus, and influenza; transthoracic echocardiogram, high-resolution computed tomography (CT) chest, ultrasound abdomen, CT abdomen, and pelvis; and bone marrow aspirate for bacterial, fungal, and mycobacterial cultures. Autoantibodies screen was also unremarkable with undetectable antinuclear antibody, extractable nuclear antigens, antidouble stranded DNA, antineutrophil cytoplasmic antibody, and rheumatoid factor.

**Figure 1:**
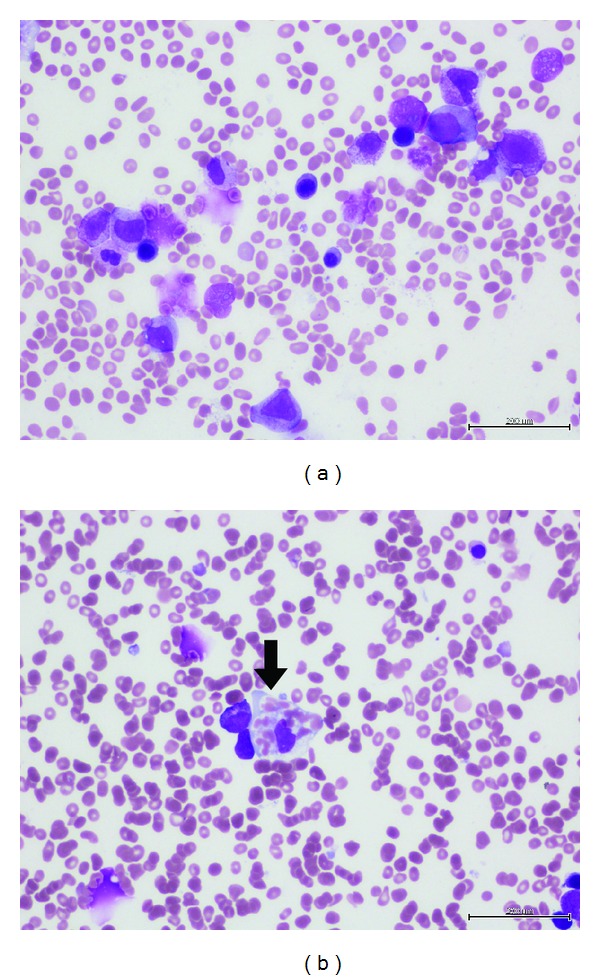
Bone marrow aspirate showed no residual CLL (a) but instead hemophagocytosis (b).

Serial bone marrow aspirate and trephine biopsies were performed and these failed to demonstrate residual CLL (Figure 1(a)). Instead, abundant histiocytes with evidence of hemophagocytosis (Figure 1(b)) were found along with increased iron stores and occasional ring sideroblasts. Given the constellation of other findings—persistent fever ≥38.5°C, splenomegaly, pancytopaenia, and hyperferritinaemia (11,605 *μ*g/L, normal range 20–500 *μ*g/L), a diagnosis of hemophagocytic lymphohistiocytosis (HLH) was made. Antibiotics were withdrawn and treatment with HLH-2004 protocol was commenced using dexamethasone and etoposide. This resulted in prompt resolution of fever and systemic symptoms. Cyclosporine was added on day 4. His ferritin levels also dropped rapidly to 5,098 *μ*g/L on day 5.

Nine days into treatment, he developed neutropenic septic shock with positive blood cultures for *Klebsiella pneumoniae *and *Clostridium difficile*. He was transferred to intensive care unit for inotropic support and treated with appropriate antimicrobials. However, he remained pancytopenic and developed refractory shock with multiorgan dysfunction syndrome. All active treatment was withdrawn after extensive discussion with the patient and family members. He deceased 13 days into starting treatment for HLH.

**Figure 2:**
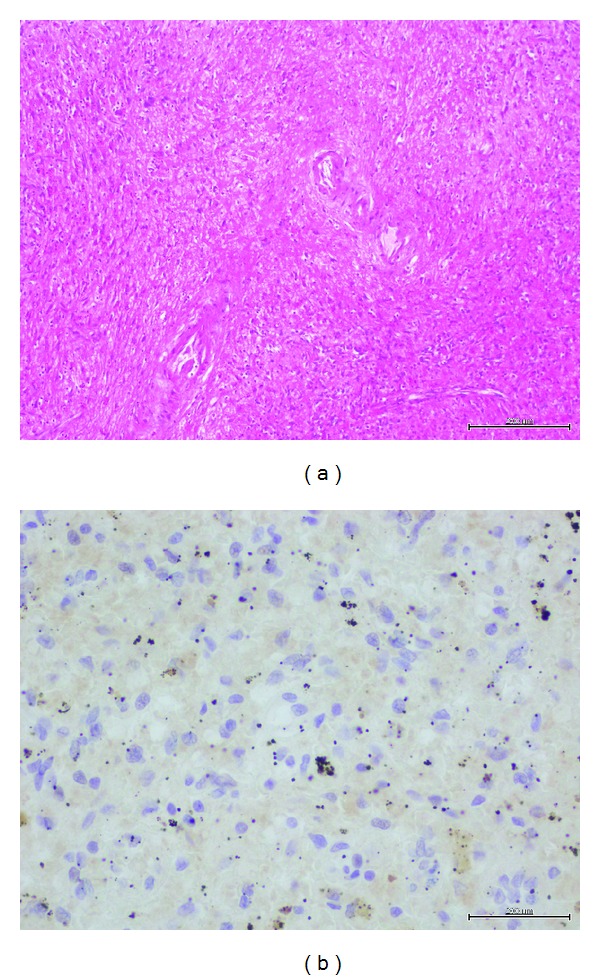
Splenic biopsy demonstrated the lack of lymphocytes (a). This is confirmed by the negative staining of CD79a (b), showing no residual B-cell population after rituximab.

**Figure 3:**
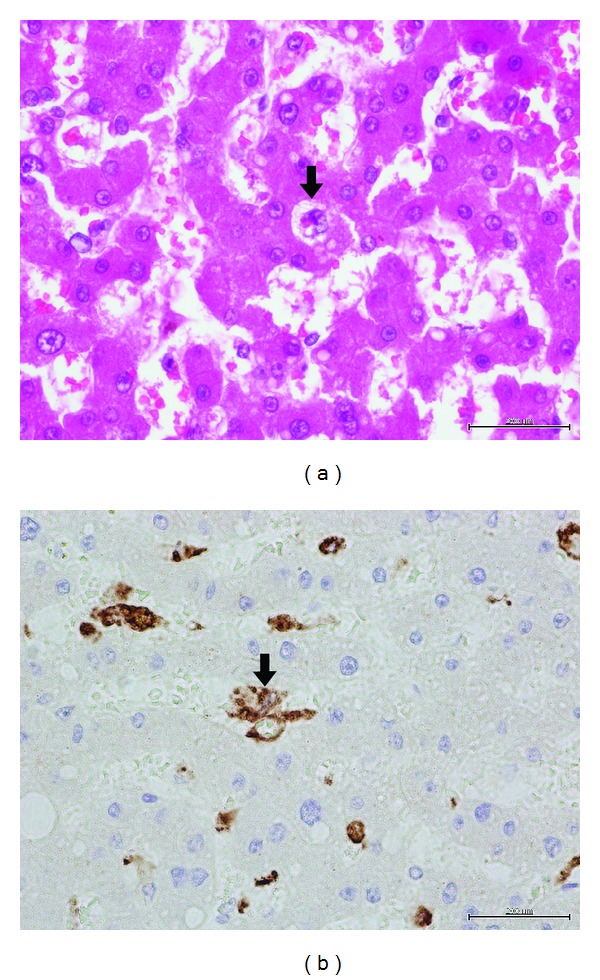
Liver biopsy showed hemophagocytosis (a) by the activated macrophage as stained by CD68 (b).

Limited post-mortem examination was performed. Splenic biopsy demonstrated the lack of lymphocytes (Figure 2(a), Hematoxylin and Eosin [H&E] stain), which was confirmed by the negative staining of CD79a (Figure 2(b)), showing no residual B-cell population after rituximab. Liver biopsy showed hemophagocytosis (Figure 3(a), H&E stain) by the activated macrophage, as stained by CD68 (Figure 3(b)). In summary, there were abundant histiocytes with hemophagocytosis without any evidence of residual CLL.

## 3. Discussion

Secondary HLH is increasingly recognized to be associated with various causes including infections, autoimmune diseases, and malignancy. Malignancy-associated HLH has been reported primarily with lymphomas or leukemias of the T- or NK-cell lineages [1]. Many have simultaneous bacterial, viral, or fungal infections that may serve as a trigger for HLH in the context of a dysfunctional immune system.

In accordance with the International Histiocyte Society guidelines [2], our patient met 5 out of 8 criteria. Soluble CD25 and NK cell activity are not routinely available in our institution. He also did not have hypertriglyceridemia or hypofibrinogenemia. Even though the criteria only require a ferritin level of >500 *μ*g/L, a level of over 10,000 *μ*g/L would have a 90% sensitivity and 96% specificity for the diagnosis of HLH [3].

Although hemophagocytosis is a hallmark of activated macrophages, it is neither sensitive nor specific for HLH and its presence should only be considered supportive [4]. It is not always present on the initial biopsy and may only be revealed on serial examinations. We illustrated this point with repeated bone marrow examination to highlight the evolution of hemophagocytosis during the course of the disease.

HLH is only rarely reported in association with CLL. Literature search revealed only 6 such cases [5–10]. At presentation with the HLH, our patient had no identified infective source despite extensive workup and multiple antimicrobials. He remained well despite persistent fever when the antimicrobials were withdrawn. This is in contrast to the other cases which were triggered by various infections including H1N1 influenza [5], EBV [6], and histoplasmosis [7, 8].

Fludarabine, given its role in suppression of cell-mediated immunity, has been linked to the development of HLH. Meki et al. [9] reported a case where a 59-year-old woman developed HLH after treatment with fludarabine and cyclophosphamide. Their conclusion, however, was that the HLH may have been present prior to initiating therapy and declared itself on progression of the CLL. Ferreira et al. [11] reported 3 children with systemic juvenile arthritis who developed HLH while undergoing fludarabine-based stem cell transplantation, although it is uncertain if fludarabine or the underlying autoimmune disease was the etiologic agent. In another case of HLH in a 75-year-old man being treated for Richter's transformation of CLL [6], this is most likely triggered by EBV infection as demonstrated on PCR, rather than the fludarabine-based chemotherapy.

To our knowledge, this is the second reported case of HLH after a rituximab-containing chemotherapy. The other reported case was in a patient with systemic lupus erythematosus and thrombotic thrombocytopenic purpura [12]. Our case is also unique in that there was no evidence of CLL disease at autopsy, a somewhat unexpected finding. This suggests that the chemotherapy may be implicated in the development of HLH, rather than a malignancy-associated secondary HLH.

Of note, adult-onset familial HLH has been reported in up to age 75 years when tested for specific mutations [13] but these genetic tests have very limited availability. We are unable to rule out the possibility of an underlying hereditary or acquired genetic predisposition.

In conclusion, our patient had no identifiable infections or residual CLL disease, raising the possibility that B-cell suppression by rituximab or T-cell suppression by fludarabine may have been the precipitating cause for HLH.
